# Detection of *Babesia* spp. and *Anaplasma* spp. in Wild Boars from Romania

**DOI:** 10.3390/ani15172542

**Published:** 2025-08-29

**Authors:** Ioan Cristian Dreghiciu, Diana Hoffman, Simona Dumitru, Tiana Florea, Mirela Imre, Tatiana Rugea, Vlad Iorgoni, Anamaria Plesko, Sorin Morariu, Ion Oprescu, Marius Stelian Ilie

**Affiliations:** 1 Department of Parasitology and Parasitic Disease, Faculty of Veterinary Medicine, University of Life Sciences “King Mihai I” from Timisoara, 119 Calea Aradului, 300645 Timisoara, Romania; diana.hoffman@usvt.ro (D.H.); sujic.tijana@yahoo.com (T.F.); mirela.imre@usvt.ro (M.I.); anamaria.plesko@usvt.ro (A.P.); sorinmorariu@usvt.ro (S.M.); ionoprescu@usvt.ro (I.O.); 2 Veterinary and Food Safety Directorate 4, Surorile Martir Caceu, 300585 Timisoara, Romania; simonagiubega@gmail.com (S.D.); rugea.tatiana-tm@ansvsa.ro (T.R.); 3 Department of Infectious Diseases and Preventive Medicine, Faculty of Veterinary Medicine, University of Life Sciences “King Mihai I” from Timisoara, 119 Calea Aradului, 300645 Timisoara, Romania; vlad.iorgoni@usvt.ro

**Keywords:** wild boar, *Babesia* spp., *Anaplasma* spp., real-time PCR, sequencing, Romania

## Abstract

While wild boars (*Sus scrofa*) can act as hosts and reservoirs for various tick-borne pathogens, including *Babesia* spp. and *Anaplasma* spp., relevant data from Romania remain scarce. This study aimed to detect these agents in blood samples collected from 321 wild boars across nine Romanian counties between 2021 and 2024. Molecular screening using real-time PCR revealed a prevalence of 6.85% for *Babesia* spp. and 9.34% for *Anaplasma* spp. Sequencing confirmed the presence of *Anaplasma phagocytophilum* in four randomly selected positive samples, and the results for *Babesia* spp. represent the first detection of these pathogens in Romanian wild boars. These findings indicate that wild boars could contribute to the maintenance of tick-borne pathogens in natural ecosystems, and might represent a source of infection for other animals or even humans. The presented data can serve as a baseline for future comparative studies and may guide targeted control measures to reduce the potential spread of tick-borne pathogens through wild boar populations in Romania. Ongoing molecular monitoring, together with more detailed species identification, will help to clarify the epidemiological relevance of these pathogens and support effective wildlife and public health strategies in Romania.

## Simple Summary

Wild boars (*Sus scrofa*) are widespread across Europe and can carry ticks that transmit diseases to animals and people. This study assessed the prevalence of two important groups of pathogens, *Babesia* spp. and *Anaplasma* spp., in wild boar blood samples collected from nine counties in Romania. Using modern molecular techniques, we found that about 7% of the animals carried *Babesia* spp. and about 9% carried *Anaplasma* spp. Further testing confirmed the presence of *Anaplasma phagocytophilum*, a known cause of disease in humans and animals. For *Babesia* spp., this is the first time that their genetic material has been identified in Romanian wild boars. These results suggest that wild boars could help to maintain these disease-causing agents in the environment, creating a risk for other animals or even people who have contact with infected ticks. Our research highlights the importance of continued monitoring of wildlife for such pathogens, in order to protect both animal and human health.

## 1. Introduction

Wild boars (*Sus scrofa*)—including Eurasian wild boars (*Sus scrofa* Linnaeus, 1758), feral pigs (*Sus scrofa domesticus*), and their hybrids—are present on every continent except Antarctica [1]. They are distributed across an extensive variety of habitats, including semi-arid zones, marshlands, forests, and alpine grasslands. In recent years, their presence has also been recorded within urban and peri-urban settings in several European cities [2].

In recent decades, wild boar populations have grown substantially throughout Europe, emerging as a considerable management challenge. After suffering a pronounced decline due to intensive hunting following the Second World War, their numbers have progressively risen, supported by favorable wildlife management [3], transformations in landscape structure [4], and climate shifts [5]. With the highest reproductive potential among ungulates [6,7] and relatively low natural mortality—mostly driven by harsh weather, disease, or predation from wolves (*Canis lupus*) [8,9,10,11]—wild boar populations have expanded markedly. This growth has led to a variety of negative consequences, such as damage to agricultural areas and human property [12], declines in plant and animal species richness and abundance [13], and issues relating to their role as reservoirs for several epizootic and zoonotic diseases, including trichinellosis and African swine fever (ASF) [14,15].

Wild animals—including wild boars—can serve as hosts for ticks which act as vectors for a broad spectrum of pathogens, including zoonotic agents such as those causing babesiosis, anaplasmosis, and ehrlichiosis. Ticks can transmit a wide variety of microorganisms, including *Borrelia* spp., *Anaplasma* spp., *Francisella* spp., *Rickettsia* spp., *Babesia* spp., the tick-borne encephalitis virus, and others [16]. Although the role of the wild boar as a tick host has been documented across numerous regions of the world [17,18,19], relevant data from Europe remain scarce. Beyond the commonly reported infestations with *Ixodes ricinus* [20,21,22], wild boars have also been occasionally found to carry *Dermacentor reticulatus* [23] and *D. marginatus* [24,25].

*Anaplasma phagocytophilum* is the most extensively investigated tick-borne pathogen in wild boar populations across Europe. Its reported prevalence in wild boars ranges from zero in countries such as Spain and Italy [26,27,28] to as high as 70.6% in Sweden [29]. Detection of *A. phagocytophilum* using various methods and target genes has also been documented in Belgium [30], Portugal [31], Slovenia [32], Romania [33], Poland [34], and Germany [21], with prevalence rates reported between 1.0% and 12.5%.

Strains harbored by deer and other wild ruminants generally belong to ecotypes adapted to cervids, which—although rarely associated with human infection—play an important role in maintaining bacteria within natural habitats and supporting the circulation of *A. phagocytophilum* among tick populations. These cervid-associated strains are considered epidemiologically distinct from those detected in dogs, horses, and wild boars, which have been linked to ecotypes with recognized zoonotic potential and involvement in human granulocytic anaplasmosis (HGA) [34,35,36,37,38]. In Europe, HGA continues to be under-recognized and under-reported, primarily because infections are frequently mild or even asymptomatic [39].

Piroplasmids pose a considerable threat to animal health and may represent a zoonotic risk. Although there is substantial research on *Babesia* infections in deer populations [40], knowledge about their presence and significance in wild boars—which have similarly high abundance—is still limited. To date, molecular investigations across Europe have failed to detect piroplasmids in wild boars from Hungary, Slovakia, Germany, and Portugal [21,23,31,41]. Only a few reports have reported piroplasmids in wild boars, including some findings of unidentified *Theileria* species in Italy and Portugal [31,42,43] and a single case of *B. bigemina* in Italy [43]. Additionally, the pathogen responsible for porcine babesiosis in domestic pigs reported from Sardinia [44] was confirmed to be absent in wild boar populations inhabiting the same area [45].

The aim of our study was to detect the presence of *Babesia* spp. and *Anaplasma* spp. in wild boar blood samples from Romania using molecular methods.

## 2. Materials and Methods

### 2.1. Sample Collection

Between October 2021 and May 2024, sampling activities were conducted at various hunting grounds located in the counties of Mureș (MS), Alba (AB), Sibiu (SB), Hunedoara (HD), Timiș (TM), Arad (AR), Caraș-Severin (CS), Mehedinți (MH), and Maramureș (MM). These efforts aimed to collect biological material—specifically, blood samples—from wild boars. Blood samples were collected on-site immediately after the animals were legally hunted during the open hunting season in authorized hunting grounds. Sampling was performed directly from the heart and abdominal cavity shortly after culling, prior to field dressing, in order to minimize degradation and ensure sample integrity. No animals were transported to separate facilities for this process. After collecting, the samples were immediately stored at a temperature of −18 °C to preserve their integrity until laboratory analysis could be performed.

It is important to highlight that all samples were collected solely from wild boars hunted in authorized hunting grounds across these counties. The wild boars included in this study were legally hunted by licensed hunters during the open hunting seasons authorized for wild boar each year, in compliance with current hunting regulations. This approach ensured both animal welfare standards and adherence to legal frameworks governing wildlife management.

### 2.2. DNA Extraction

For this study, the extraction of DNA was carried out with the PureLink Genomic DNA Mini Kit (Invitrogen, Carlsbad, CA, USA) in accordance with the manufacturer’s recommendations to preserve sample integrity. Once isolated, the DNA was stored in Eppendorf tubes at −18 °C until further amplification procedures targeting specific pathogens were performed.

### 2.3. Molecular Analysis for Babesia spp. and Anaplasma spp.

Detection of both pathogens was performed using a real-time PCR assay, amplifying a 116 bp specific segment of the 18S rRNA gene for *Babesia* spp. [46] and a 160 bp fragment of the 16S rRNA gene for *Anaplasma* spp. [47]. For detecting *Babesia* spp. a specific TaqMan probe (Bab18S-p: FAM-AAGTCATCAGCTTGTGCAGATTACGTCCCT-BHQ1), in combination with a forward primer (Bab18S-f: 5′-CATGAACGAGGAATGCCTAGTATG-3′) and a reverse primer (Bab18S-r: 5′-CCGAATAATT CACCGGATCACTC-3′) was employed. For *Anaplasma* spp. detection, a specific primer pair—Ana_spp_16S_F (5′-CTTAGGGTTGTAAAACTCTTTCAG-3′) and Ana_spp_16S_R (5′-CTTTAACTTACCAAACCGCCTAC-3′)—in conjunction with a TaqMan MGB probe, Ana_spp_16S_P (5′-ATGCCCTTTACGCCCAATAATTCCGAACA-3′) (ThermoFisher Scientific, Pleasanton, CA, USA) was used; these oligonucleotides were selected based on their high sensitivity and specificity, following the methodology outlined by Boularias et al. [48]. The reaction was carried out in a final volume of 25 μL, containing 8.5 μL template, 1 μL forward primer (10 μM), 1 μL reverse primer (10 μM), 1 μL probe (3 μM), 1 μL enzyme, and 12.5 μL buffer. Amplification was performed on a QuantStudio 7 Flex platform (Applied Biosystems, Pleasanton, CA, USA) with the following thermal profile: 48 °C for 10 min, 95 °C for 10 min, followed by 40 cycles of denaturation at 95 °C for 15 s and annealing/extension at 60 °C for 45 s. In the absence of a limit of detection established using a standardized or quantified positive control, a sample was considered positive for *Babesia* spp. if it showed a Ct value below 39, accompanied by a specific amplification curve characteristic of true-positive reactions. To validate the assay, each PCR run included a positive control consisting of a confirmed *Babesia caballi* sample, as well as two negative controls: an extraction negative control (ME) and a negative template control (NTC).

In addition, each run performed for *Anaplasma* detection included a positive control represented by a sample previously confirmed as *Anaplasma* spp., as well as two negative controls: an extraction negative control (ME) and a negative template control (NTC).

### 2.4. Conventional PCR

As part of the molecular analysis, conventional PCR was applied to characterize samples that tested positive for *Babesia* spp. and *Anaplasma* spp. Specifically, for *Anaplasma* spp., the primers Ana23S-212f (5′-ATAAGCTGCGGGGAATTGTC-3′) and Ana23S-908r (5′-GTAACAGGTTCGGTCCTCCA-3′) were used to target a 23S rRNA gene fragment of approximately 500 bp [48].

To detect *Babesia* spp., amplification focused on the 18S rRNA gene, employing the primers BabsppF1 (5′-GTTTCTGMCCCATCAGCTTGAC-3′) and BabsppR (5′-CAAGACAAAAGTCTGCTTGAAAC-3′), which produced fragments between 422 and 440 bp [49]. These primer pairs were chosen to support reliable sequencing and phylogenetic interpretation of the products.

PCR reactions were prepared using a commercial master mix MyTaq Red Mix (BIOLINE, London, UK), specific primers, and DNA template, and were carried out on a MyCycler thermal cycler (Bio-Rad, Berkeley, CA, USA) following a standard amplification protocol that included denaturation, annealing, and extension steps, with final cooling to preserve the products [48].

The amplified products were then resolved through horizontal electrophoresis on a 1.5% agarose gel stained with RedSafe (iNtRON Biotechnology, Seongnam-si, Gyeonggi-do, Republic of Korea).

Selected amplicons were purified using the α + Solution GEL/PCR Purification Kit (Alphagen, Changzhi, Taiwan), following the manufacturer’s protocol. Sanger sequencing was performed on both strands by Macrogen Europe B.V. (Amsterdam, The Netherlands). After sequencing, data were edited for quality and compared to GenBank entries using the BLAST version 5 platform [50] to confirm the identity of the pathogens.

Sequencing analysis revealed that all four *Anaplasma* spp. samples corresponded to *Anaplasma phagocytophilum*, confirming the specificity of the real-time PCR assays and demonstrating the presence of this zoonotic bacterium among wild boars.

The sequencing results obtained from Macrogen for the *Anaplasma phagocytophilum* samples were submitted to GenBank under the following accession numbers: PV876746, PV876747, PV876748, and PV876749.

Phylogenetic analyses were performed using Phylogeny.fr “one-click analysis” (https://www.phylogeny.fr/, accessed on 2 August 2025), and sequences were aligned with ClustalW (v2.1). After alignment, ambiguous regions (i.e., containing gaps and/or poorly aligned) were removed with Gblocks (v0.91b). The phylogenetic tree was reconstructed using the maximum likelihood method implemented in the PhyML program (v 3.1/3.0 aLRT, https://www.phylogeny.fr/, accessed on 24 March 2025). The default substitution model was selected, assuming an estimated proportion of invariant sites (of 0.000) and 4 gamma-distributed rate categories to account for rate heterogeneity across sites. The gamma shape parameter was estimated directly from the data (gamma = 2.391). Reliability for internal branches was assessed using the aLRT test (SH-Like). The graphical representation and editing of the phylogenetic tree were performed with TreeDyn (v198.3) [51].

### 2.5. Statistical Analysis

Statistical analyses were conducted using EPI Info v.7.2.7.0 (CDC, Atlanta, GA, USA, 2025). To compare differences in positivity, both across counties and between pathogens, two-tailed Fisher’s exact tests were performed. A *p*-value less than or equal to 0.05 was considered statistically significant.

## 3. Results

### 3.1. Sampling and Detection of Babesia spp. and Anaplasma spp. in Wild Boars via Real-Time PCR

**Figure 1:**
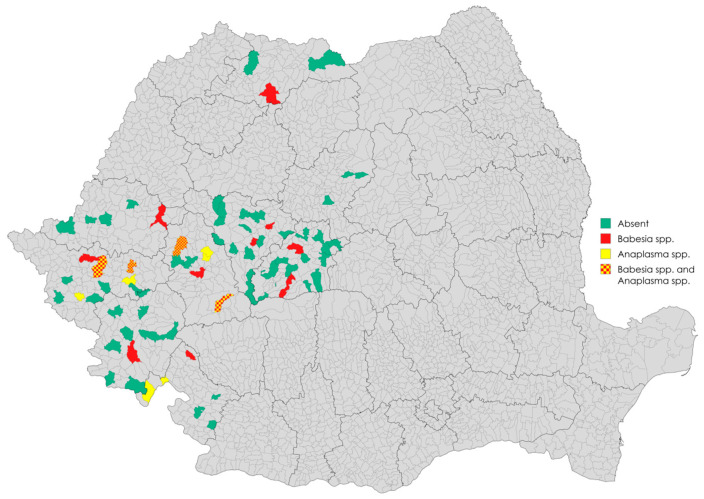
Spatial distribution of *Babesia* spp. and *Anaplasma* spp. across hunting grounds in Romania [52]. Legend: green—absence in the respective hunting ground; red—presence of *Babesia* spp.; yellow—presence of *Anaplasma* spp.; yellow and red—presence of both *Babesia* spp. and *Anaplasma* spp.

Over a 4-year sampling period, a total of 321 wild boar blood samples were collected from nine counties across Romania. The samples covered a representative geographic area and a diverse range of hunting grounds (Figure 1).

Detailed information on the number of hunting grounds, positivity, and distribution of collected blood samples within each county is provided in Table 1.

**Figure 2:**
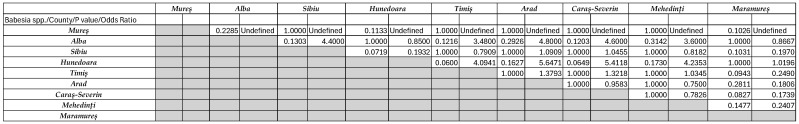
Statistical analysis using Fisher’s exact test of the samples studied to identify *Babesia* spp.

**Figure 3:**
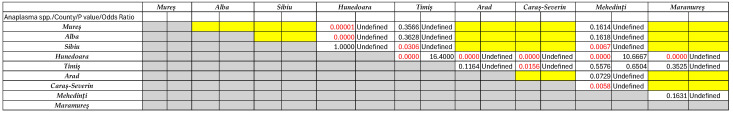
Statistical analysis using Fisher’s exact test of the samples studied to identify *Anaplasma* spp. Legend: yellow = values are unavailable and impossible to calculate due to a lack of positivity; red = statistically significant differences between the different counties.

Statistical analysis of the values obtained through the processing of samples from wild boars for the presence of *Babesia* spp. did not indicate statistically significant differences (Figure 2). However, statistically significant differences were identified when analyzing the results obtained for the identification of *Anaplasma* spp. between the counties of Mures and Alba vs. Hunedoara; Sibiu vs. Timis and Mehedinți; Hunedoara vs. Timis, Arad, Caraș Severin, Mehedinți, and Maramureș; Timis vs. Caraș Severin; and Arad vs. Mehedinți (Figure 3).

The obtained amplification curves revealed specific signals with Ct values ranging from 32.6 to 39, indicating the presence of low amounts of parasitic DNA within the tested samples. In the absence of a limit of detection established using a quantified standard, reactions that exhibited a specific amplification curve and Ct values below 39 were interpreted as positive, indicating the presence of low amounts of parasitic DNA.

Of the 321 blood samples analyzed via real-time PCR, 30 (9.34%) tested positive for *Anaplasma* spp.

Several samples yielded Ct values ranging from 27 to 30 (e.g., samples 81, 92, and 97), while additional positive detections were observed with Ct values between 31 and 36. These results fall within the accepted range for true-positive reactions and support the reliability of *Anaplasma* spp. detection across the tested samples.

These results collectively confirm the presence of *Anaplasma* spp. in wild boar populations, highlighting their potential role as a reservoir for tick-borne pathogens and emphasizing the value of molecular surveillance for assessing epidemiological risks in wildlife.

### 3.2. Detection and Sequencing of Babesia spp. and Anaplasma spp. Via Conventional PCR

In order to achieve species-level identification of the detected pathogens, four *Babesia* spp.-positive and four *Anaplasma* spp.-positive samples were randomly selected from the real-time PCR-positive results and subjected to conventional PCR. The resulting amplicons were subsequently purified and sent for sequencing to Macrogen Europe (The Netherlands). This random selection strategy aimed to provide a representative overview of the pathogen species circulating in the tested wild boar population.

The analyzed sequences exhibited 100% identity and full query coverage with multiple complete genomes of *A. phagocytophilum* (Table 2), including the strains Norway variant1 (CP046639.1), SLO-1 (CP166491.1), and Norway variant2 (CP015376.1).

Unfortunately, for *Babesia* spp., none of the samples yielded interpretable sequencing results.

**Figure 4:**
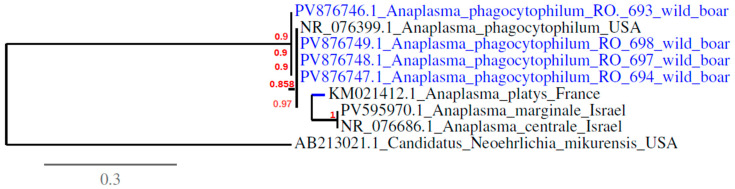
Phylogenetic relationships between *Anaplasma* spp. detected in wild boars in this study (highlighted in blue) and other *Anaplasma* spp. available in GenBank, determined via ML analysis of partial sequences of 23S rRNA. *Candidatus Neoehrlichia mikurensis* (AB213021.1) was set as an outgroup.

A phylogenetic tree illustrating the relationships among *Anaplasma* spp. identified in this study is shown in Figure 4. The tree was constructed based on 23S rRNA gene sequences, employing the maximum likelihood (ML) method. The Romanian *A. phagocytophilum* isolates obtained from wild boars and newly deposited in GenBank (accession numbers: PV876746.1, PV876747.1, PV876748.1, PV876749.1) clustered closely with a reference strain from the USA, indicating a high degree of genetic similarity. Phylogenetic reconstruction clearly separated these isolates from other *Anaplasma* spp., including *A. platys*, *A. marginale*, and *A. centrale*, with *Candidatus Neoehrlichia mikurensis* serving as an outgroup to root the tree.

## 4. Discussion

Of the 321 blood samples collected and tested via real-time PCR, 22 (6.85%) were positive for *Babesia* spp., indicating a detectable circulation of these pathogens within the studied wild boar population and confirming their presence in the local population. Sequencing results validated the presence of *A. phagocytophilum* in a subset of positive samples, demonstrating its notable presence among the considered wild boar population; in contrast, no species-level data were obtained for *Babesia* spp.

The obtained results strongly support the precise identification of the detected organism as *A. phagocytophilum* and confirm its high genetic conservation within this group.

Few studies have explored *Babesia* and *Anaplasma* simultaneously in wild boars and, for Romanian populations, new data on this subject remain scarce.

These findings also support the circulation of *Babesia* species in the studied wild boar populations, providing valuable information regarding the potential risk of vector-borne transmission in wildlife ecosystems. The high Ct values observed in some samples may be influenced by factors such as sample quality, partial DNA degradation during handling or storage, and/or the presence of endogenous PCR inhibitors commonly found in blood samples, such as hemoglobin or other cellular components. Several investigations have assessed the occurrence of *A. phagocytophilum* and *Babesia* spp. in wild boars from various areas of Romania. Kiss et al. carried out an extensive survey between 2007 and 2012, analyzing 870 tissue samples (liver, spleen, and kidney) collected from wild boars in 16 counties. They reported a prevalence of 4.48% for *A. phagocytophilum* detected via nested PCR targeting the 16S rRNA gene, with most positive cases recorded in central Transylvania [33]. More recently, Matei et al. investigated 203 blood samples gathered during the 2019–2020 and 2020–2021 hunting seasons in Sălaj County, identifying *A. phagocytophilum* in 3% of samples through conventional and nested PCR approaches focused on the 16S rRNA and groEL genes, while no positives were found for *Babesia* spp. [39]. In 2023, Dreghiciu et al. also examined 29 blood samples from wild boars in two counties in Romania, detecting one positive case of *A. phagocytophilum* (3.44%) using PCR amplification of the *epank1* gene [53]. Although these studies reported relatively low prevalence figures, they all confirmed the presence of *A. phagocytophilum* in wild boar populations across several Romanian regions.

These results underscore the importance of continuous molecular monitoring to better understand the involvement of wildlife in the epidemiology of tick-borne infections. Our results contribute to a better understanding of the occurrence of *Babesia* spp. and *Anaplasma* spp. in wild boars from Romania, as well as their relevance in the context of host–parasite interactions and potential zoonotic risks. The detection of these pathogens in animals suggests that wild boars may act as potential reservoirs, maintaining these agents in the ecosystem and potentially facilitating their transmission to domestic animals and humans in overlapping habitats. Even in the absence of clinical signs, such infections may influence the health and population dynamics of animals over time. Considering that *Anaplasma* spp. include species of zoonotic importance, our findings underline the need to integrate wildlife pathogen monitoring into broader surveillance programs. Such data are essential for informing evidence-based decisions in wildlife management and supporting preventive measures within a One Health framework. In Belgium, *A. phagocytophilum* was detected in only 0.97% of wild boar spleen samples (5 out of 513)—a prevalence much lower than the 85.6% found in roe deer from the same area [30]. Sequencing confirmed that the species responsible for wild boar infections belonged to strains previously identified in local deer populations, suggesting possible spillover between hosts.

In contrast, the repeated absence of *Babesia* spp. in previous tested samples may suggest either their limited distribution in wild boars or potentially reduced host susceptibility; however, more extensive research is warranted to clarify these aspects.

Compared to these previous investigations in Romania, our study highlights a higher prevalence of *Anaplasma* spp. (9.34%) and, importantly, documents the presence of *Babesia* spp. DNA (6.85%) in wild boar blood samples, which has not previously been reported in Romania. This suggests regional or temporal differences in pathogen circulation, potentially influenced by factors such as local vector abundance, habitat changes, or the wild boar population density, underscoring the need for continued monitoring efforts at a broader scale.

A study conducted by Defaye et al. (2021) in Corsica analyzed 158 wild boars and identified three samples positive for *Babesia* spp., although sequencing did not confirm the exact species involved. In the same study, a very low prevalence of *Anaplasma* spp. was reported, with only 0.88% positivity (10 out of 113 samples) [54]. These results indicate that both *Anaplasma* and *Babesia* may circulate among wild boars and their associated tick populations in France, but with considerable regional variation.

Multiple surveys carried out across Italy between 2010 and 2022 have highlighted that wild boars can harbor both *Anaplasma* and *Babesia* species, although with variable findings depending on region.

In the south of the country, Sgroi et al. (2023) analyzed 243 spleen samples obtained between 2016 and 2022, detecting *Babesia vulpes* in 5.3% and *Babesia capreoli* in 0.9%, for a total prevalence of 6.2% [55]. In sharp contrast, investigations performed by Zobba et al. (2014) in Sardinian wild boars did not reveal the presence of any *Babesia* spp. among 52 blood samples, underlining pronounced regional differences between mainland Italy and its islands [45].

Moving north, Zanet et al. (2014) screened over a thousand wild ungulates, including 257 wild boars, and confirmed the presence of *Babesia bigemina* in 4.67% of the wild boar subset through nested PCR and sequencing [43].

In central Italy, Ebani et al. (2017) examined 100 spleen samples collected during several hunting seasons between 2013 and 2015 and reported a single case of *A. phagocytophilum* infection (1%), marking the first documented detection of this pathogen in Italian wild boars [56].

Across Europe, the reported prevalence of *A. phagocytophilum* infection in wild boar populations has shown considerable variation, with figures ranging from 0% [57] to as high as 70.6% [29]. Higher infection rates have been documented in southern Germany, where 12.5% of wild boars tested positive for *A. phagocytophilum* [21], as well as in Poland (20.34%) [58], Slovakia (28.2%) [23], and Sweden, where a remarkably high prevalence of 70.6% was noted [29]. Conversely, surveys carried out in Austria and Spain did not detect any infected wild boars at all [27,57,59]. This wide range of prevalence between countries may reflect differences in climate, habitat, tick species distribution, wild boar population densities, or even the sampling methods and molecular targets used by researchers [40].

These findings highlight the complex epidemiology of *A. phagocytophilum* in wildlife hosts across Europe and the need for harmonized monitoring to better understand the role of wild boars in the transmission cycle of this zoonotic pathogen.

In a similar way, the circulation of *Babesia* spp. in wild boar populations—so far observed with a very low (or zero) prevalence, and rarely investigated in previous research—highlights the need for further studies to determine whether these animals play a meaningful role in sustaining and spreading such protozoan infections in European wildlife.

One of the limitations of this study was the inability to achieve species-level identification of *Babesia* due to the unsuccessful sequencing of positive samples. This outcome was likely influenced by the low amount of target DNA and the presence of inhibitors in blood samples, as well as possible mismatches in the primers used. To overcome these challenges, future research should consider alternative strategies such as nested or semi-nested PCR protocols to increase sensitivity, the use of species-specific primers targeting highly conserved genomic regions, and improved DNA purification methods to minimize inhibitory compounds.

Differences in sample size among counties—particularly in areas with limited available samples (e.g., Maramureș)—may influence the reliability of prevalence estimates and limit the ability to detect true spatial patterns. Future studies should aim for more balanced sampling to improve statistical power and the robustness of regional comparisons. These results are expected to be valuable for clarifying the possible roles of wild boars as potential hosts for tick-borne pathogens, with consequences for both animal and human health. Overall, this study supports the importance of continued molecular monitoring and expanded investigations to better understand the epidemiological patterns of such pathogens in wildlife populations.

## 5. Conclusions

This study provides new insights into the presence of *Babesia* spp. and *Anaplasma* spp. in wild boar populations from Romania. Using molecular methods, *Babesia* spp. DNA was detected in 6.85% of the tested samples while *Anaplasma* spp. was identified in 9.34% of the tested samples, with sequencing confirming *A. phagocytophilum* in a subset of positive samples.

Although the prevalence of *Babesia* spp. was low and species-level confirmation could not be achieved, the findings highlight the circulation of these pathogens among Romanian wild boars, representing the first detection of *Babesia* spp. at the genus level in wild boars in Romania.

## Figures and Tables

**Table 1:** Distribution of wild boar blood samples by county, number of hunting grounds, and real-time PCR positivity.

County	Hunting Grounds	Nr. of Samples	Positivity	
			*Babesia* spp.	*Anaplasma* spp.
Mureș	2	17	0 (0%)	0 (0%)
Alba	14	18	3 (16.6%)	0 (0%)
Sibiu	14	46	2 (4.3%)	0 (0%)
Hunedoara	7	21	4 (19%)	14 (66.7%)
Timiș	10	92	5 (5.4%)	10 (10.9%)
Arad	5	25	1 (4%)	0 (0%)
Caraș-Severin	9	48	2 (4.2%)	0 (0%)
Mehedinți	6	38	2 (5.3%)	6 (15.8%)
Maramureș	3	16	3 (18.8%)	0 (0%)
Total	70	321	22 (6.9%)	30 (9.3%)

**Table 2:** Characteristics of wild boar blood samples identified as positive for *A. phagocytophilum.*

Sample Number	Date	Ct	County	Hunting Ground	Age	Positivity
81	12 January 2022	27.650	Timiș	42 Buziaș	12	*A. phagocytophilum*
127	17 February 2022	33.154	Hunedoara	20 Bobâlna	30	*A. phagocytophilum*
08	17 February 2022	38.350	Hunedoara	16 Băița	16	*A. phagocytophilum*
258	15 December 2023	33.847	Mehedinți	02 Dubova	16	*A. phagocytophilum*

Legend: Age refers to the age of wild boars expressed in months, abbreviated for spatial optimization of the table. Samples are listed in chronological order by collection date.

## Data Availability

Data are contained within the article.

## References

[1] Ruiz-Fons F. (2017). A Review of the Current Status of Relevant Zoonotic Pathogens in Wild Swine (*Sus scrofa*) Populations: Changes Modulating the Risk of Transmission to Humans. Transbound. Emerg. Dis..

[2] Licoppe A., Prevot C., Heymans M., Bovy C., Casaer J., Cahill S. (2013). Wild Boar/Feral Pig in (Peri-)Urban Areas. Managing Wild Boar in Human-Dominated Landscapes, Proceedings of the International Union of Game Biologists—Congress IUGB.

[3] Maillard D., Gaillard J.M., Hewison M., Ballon P., Duncan P., Loison A., Toïgo C.E.B., Bonenfant C., Garel M., Apollonio M., Andersen R., Putman R. (2010). Ungulates and Their Management in France. European Ungulates and Their Management in the 21st Century.

[4] Morelle K., Fattebert J., Mengal C., Lejeune P. (2016). Invading or Recolonizing? Patterns and Drivers of Wild Boar Population Expansion into Belgian Agroecosystems. Agric. Ecosyst. Environ..

[5] Markov N., Pankova N., Morelle K. (2019). Where Winter Rules: Modeling Wild Boar Distribution in Its North-Eastern Range. Sci. Total Environ..

[6] Bieber C., Ruf T. (2005). Population Dynamics in Wild Boar *Sus scrofa*: Ecology, Elasticity of Growth Rate and Implications for the Management of Pulsed Resource Consumers. J. Appl. Ecol..

[7] Holland E.P., Burrow J.F., Dytham C., Aegerter J.N. (2009). Modelling with Uncertainty: Introducing a Probabilistic Framework to Predict Animal Population Dynamics. Ecol. Modell..

[8] Jędrzejewski W., Jędrzejewska B., Okarma H., Ruprecht A.L. (1992). Wolf Predation and Snow Cover as Mortality Factors in the Ungulate Community of the Białowieża National Park, Poland. Oecologia.

[9] Massei G., Kindberg J., Licoppe A., Gačić D., Šprem N., Kamler J., Baubet E., Hohmann U., Monaco A., Ozoliņš J. (2015). Wild Boar Populations Up, Numbers of Hunters Down? A Review of Trends and Implications for Europe. Pest Manag. Sci..

[10] Nores C., Llaneza L., Álvarez A. (2008). Wild Boar *Sus scrofa* Mortality by Hunting and Wolf *Canis lupus* Predation: An Example in Northern Spain. Wildl. Biol..

[11] Okarma H., Jędrzejewska B., Jędrzejewski W., Krasiński Z.A., Miłkowski L. (1995). The Roles of Predation, Snow Cover, Acorn Crop, and Man-Related Factors on Ungulate Mortality in Białowieża Primeval Forest, Poland. Acta Theriol..

[12] Lombardini M., Meriggi A., Fozzi A. (2017). Factors Influencing Wild Boar Damage to Agricultural Crops in Sardinia (Italy). Curr. Zool..

[13] Oja R., Soe E., Valdmann H., Saarma U. (2017). Non-Invasive Genetics Outperforms Morphological Methods in Faecal Dietary Analysis, Revealing Wild Boar as a Considerable Conservation Concern for Ground-Nesting Birds. PLoS ONE.

[14] Gavier-Widén D., Ståhl K., Neimanis A.S., Hård Av Segerstad C., Gortázar C., Rossi S., Kuiken T. (2015). African Swine Fever in Wild Boar in Europe: A Notable Challenge. Vet. Rec..

[15] Gortázar C., Ferroglio E., Höfle U., Frölich K., Vicente J. (2007). Diseases Shared between Wildlife and Livestock: A European Perspective. Eur. J. Wildl. Res..

[16] Boulanger N., Boyer P., Talagrand-Reboul E., Hansmann Y. (2019). Ticks and Tickborne Diseases. Med. Mal. Infect..

[17] Lim F.S., Khoo J.J., Tan K.K., Zainal N., Loong S.K., Khor C.S., AbuBakar S. (2020). Bacterial Communities in *Haemaphysalis*, *Dermacentor* and *Amblyomma* Ticks Collected from Wild Boar of an Orang Asli Community in Malaysia. Ticks Tick Borne Dis..

[18] Masatani T., Hayashi K., Andoh M., Tateno M., Endo Y., Asada M., Kusakisako K., Tanaka T., Gokuden M., Hozumi N. (2017). Detection and Molecular Characterization of *Babesia*, *Theileria*, and *Hepatozoon* Species in Hard Ticks Collected from Kagoshima, the Southern Region in Japan. Ticks Tick Borne Dis..

[19] Merrill M.M., Boughton R.K., Lord C.C., Sayler K.A., Wight B., Anderson W.M., Wisely S.M. (2018). Wild Pigs as Sentinels for Hard Ticks: A Case Study from Southcentral Florida. Int. J. Parasitol. Parasites Wildl..

[20] Honig V., Carolan H.E., Vavruskova Z., Massire C., Mosel M.R., Crowder C.D., Rounds M.A., Ecker D.J., Ruzek D., Grubhoffer L. (2017). Broad-Range Survey of Vector-Borne Pathogens and Tick Host Identification of *Ixodes ricinus* from Southern Czech Republic. FEMS Microbiol. Ecol..

[21] Silaghi C., Pfister K., Overzier E. (2014). Molecular Investigation for Bacterial and Protozoan Tick-Borne Pathogens in Wild Boars (*Sus scrofa*) from Southern Germany. Vector-Borne Zoonotic Dis..

[22] Skotarczak B., Adamska M., Sawczuk M., Maciejewska A., Wodecka B., Rymaszewska A. (2008). Coexistence of Tick-Borne Pathogens in Game Animals and Ticks in Western Poland. Vet. Med..

[23] Kazimírová M., Hamsíková Z., Špitalská E., Minichová Ľ., Mahríková L., Caban R., Sprong H., Fonville M., Schnittger L., Kocianová E. (2018). Diverse Tick-Borne Microorganisms Identified in Free-Living Ungulates in Slovakia. Parasit. Vectors.

[24] Maioli G., Pistone D., Bonilauri P., Pajoro M., Barbieri I., Patrizia M., Vicari N., Dottori M. (2012). Ethiological Agents of Rickettsiosis and Anaplasmosis in Ticks Collected in Emilia-Romagna Region (Italy) during 2008 and 2009. Exp. Appl. Acarol..

[25] Ortuño A., Quesada M., López S., Miret J., Cardeñosa N., Castella J., Antón E., Segura F. (2006). Prevalence of *Rickettsia slovaca* in *Dermacentor marginatus* Ticks Removed from Wild Boar (*Sus scrofa*) in Northeastern Spain. Ann. N. Y. Acad. Sci..

[26] de la Fuente J., Naranjo V., Ruiz-Fons F., Höfle U., Fernández de Mera I.G., Villanúa D., Almazán C., Torina A., Caracappa S., Kocan K.M. (2005). Potential Vertebrate Reservoir Hosts and Invertebrate Vectors of *Anaplasma marginale* and *A. phagocytophilum* in Central Spain. Vector-Borne Zoonotic Dis..

[27] Portillo A., Pérez-Martínez L., Santibáñez S., Santibáñez P., Palomar A.M., Oteo J.A. (2011). *Anaplasma* spp. in Wild Mammals and *Ixodes ricinus* from the North of Spain. Vector-Borne Zoonotic Dis..

[28] Torina A., Alongi A., Naranjo V., Scimeca S., Nicosia S., Di Marco V., Caracappa S., Kocan K.M., de la Fuente J. (2008). Characterization of *Anaplasma* Infections in Sicily, Italy. Ann. N. Y. Acad. Sci..

[29] Fabri N.D., Sprong H., Hofmeester T.R., Heesterbeek H., Donnars B.F., Widemo F., Ecke F., Cromsigt J.P.G.M. (2021). Wild Ungulate Species Differ in Their Contribution to the Transmission of *Ixodes ricinus*-Borne Pathogens. Parasit. Vectors.

[30] Nahayo A., Bardiau M., Volpe R., Pirson J., Paternostre J., Fett T., Linden A. (2014). Molecular Evidence of *Anaplasma phagocytophilum* in Wild Boar (*Sus scrofa*) in Belgium. BMC Vet. Res..

[31] Pereira A., Parreira R., Nunes M., Casadinho A., Vieira M.L., Campino L., Maia C. (2016). Molecular Detection of Tick-Borne Bacteria and Protozoa in Cervids and Wild Boars from Portugal. Parasit. Vectors.

[32] Smrdel K.S., Bidovec A., Malovrh T., Petrovec M., Duh D., Zupanc T.A. (2009). Detection of *Anaplasma phagocytophilum* in Wild Boar in Slovenia. Clin. Microbiol. Infect..

[33] Kiss T., Cadar D., Krupaci F.A., Bordeanu A.D., Spînu M. (2014). Prevalence of *Anaplasma phagocytophilum* Infection in European Wild Boar (*Sus scrofa*) Populations from Transylvania, Romania. Epidemiol. Infect..

[34] Michalik J., Stańczak J., Cieniuch S., Racewicz M., Sikora B., Dabert M. (2012). Wild Boars as Hosts of Human-Pathogenic *Anaplasma phagocytophilum* Variants. Emerg. Infect. Dis..

[35] Huhn C., Winter C., Wolfsperger T., Wüppenhorst N., Strašek Smrdel K., Skuballa J., Pfäffle M., Petney T., Silaghi C., Dyachenko V. (2014). Analysis of the Population Structure of *Anaplasma phagocytophilum* Using Multilocus Sequence Typing. PLoS ONE.

[36] Scharf W., Schauer S., Freyburger F., Petrovec M., Schaarschmidt-Kiener D., Liebisch G., Runge M., Ganter M., Kehl A., Dumler J.S. (2011). Distinct Host Species Correlate with *Anaplasma phagocytophilum* ankA Gene Clusters. J. Clin. Microbiol..

[37] Smrdel K.S., Petrovec M., Furlan S.L., Županc T.A. (2012). The Sequences of groESL Operon of *Anaplasma phagocytophilum* among Human Patients in Slovenia. FEMS Immunol. Med. Microbiol..

[38] Smrdel K.S., von Loewenich F.D., Petrovec M., Županc T.A. (2015). Diversity of ankA and msp4 Genes of *Anaplasma phagocytophilum* in Slovenia. Ticks Tick Borne Dis..

[39] Matei I.A., Estrada-Peña A., Cutler S.J., Vayssier-Taussat M., Varela-Castro L., Potkonjak A., Zeller H., Mihalca A.D. (2019). A Review on the Eco-Epidemiology and Clinical Management of Human Granulocytic Anaplasmosis and Its Agent in Europe. Parasit. Vectors.

[40] Hrazdilová K., Rybarová M., Siroký P., Votýpka J., Zintl A., Burgess H., Steinbauer V., Zakovčík V., Modrý D. (2020). Diversity of *Babesia* spp. in Cervid Ungulates Based on the 18S rDNA and Cytochrome c Oxidase Subunit I Phylogenies. Infect. Genet. Evol..

[41] Hornok S., Sugár L., Fernández de Mera I.G., de la Fuente J., Horváth G., Kovács T., Micsutka A., Gönczi E., Flaisz B., Takács N. (2018). Tick- and Fly-Borne Bacteria in Ungulates: The Prevalence of *Anaplasma phagocytophilum*, Haemoplasmas and Rickettsiae in Water Buffalo and Deer Species in Central Europe, Hungary. BMC Vet. Res..

[42] Tampieri M.P., Galuppi R., Bonoli C., Cancrini G., Moretti A., Pietrobelli M. (2008). Wild Ungulates as *Babesia* Hosts in Northern and Central Italy. Vector-Borne Zoonotic Dis..

[43] Zanet S., Trisciuoglio A., Bottero E., De Mera I.G.F., Gortázar C., Carpignano M.G., Ferroglio E. (2014). Piroplasmosis in Wildlife: *Babesia* and *Theileria* Affecting Free-Ranging Ungulates and Carnivores in the Italian Alps. Parasit. Vectors.

[44] Zobba R., Parpaglia M.L.P., Spezzigu A., Pittau M., Alberti A. (2011). First Molecular Identification and Phylogeny of a *Babesia* sp. from a Symptomatic Sow (*Sus scrofa* Linnaeus 1758). J. Clin. Microbiol..

[45] Zobba R., Nuvoli A.M., Sotgiu F., Lecis R., Spezzigu A., Dore G.M., Masia M.A., Cacciotto C., Parpaglia M.L.P., Dessì D. (2014). Molecular Epizootiology and Diagnosis of Porcine Babesiosis in Sardinia, Italy. Vector-Borne Zoonotic Dis..

[46] Racewicz M. (2015). Detection and quantification of *Anaplasma phagocytophilum* and *Babesia* spp. in *Ixodes ricinus* ticks from urban and rural environment, northern Poland, by real-time polymerase chain reaction. Exp. Appl. Acarol..

[47] Boularias G., Azzag N., Galon C., Šimo L., Boulouis H.-J., Moutailler S. (2021). High-Throughput Microfluidic Real-Time PCR for the Detection of Multiple Microorganisms in Ixodid Cattle Ticks in Northeast Algeria. Pathogens.

[48] Dahmani M., Marié J.L., Mediannikov O., Raoult D., Davoust B. (2015). First Identification of *Anaplasma platys* in the Blood of Dogs from French Guiana. Vector-Borne Zoonotic Dis..

[49] Lempereur L., Beck R., Fonseca I., Marques C., Duarte A., Santos M., Zúquete S., Gomes J., Walder G., Domingos A. (2017). Guidelines for the Detection of *Babesia* and *Theileria* Parasites. Vector-Borne Zoonotic Dis..

[50] Basic Local Alignment Search Tool (BLAST). https://blast.ncbi.nlm.nih.gov.

[51] Dereeper A., Guignon V., Blanc G., Audic S., Buffet S., Chevenet F., Dufayard J.F., Guindon S., Lefort V., Lescot M. (2008). Phylogeny.fr: Robust Phylogenetic Analysis for the Non-Specialist. Nucleic Acids Res..

[52] MapChart. https://www.mapchart.net/romania.html.

[53] Dreghiciu I.C., Imre M., Oprescu I., Florea T., Ghilean B.M., Sîrbu B.A.M., Iorgoni V., Badea C., Giubega S., Mederle N. (2023). Molecular Detection of *Anaplasma phagocytophilum* in Wild Boar (*Sus scrofa*) from Hunedoara and Timiș Counties—Preliminary Study. Rev. Rom. Med. Vet..

[54] Defaye B., Moutailler S., Pietri C., Galon C., Grech-Angelini S., Pasqualini V., Quilichini Y. (2021). Molecular Detection of Zoonotic and Non-Zoonotic Pathogens from Wild Boars and Their Ticks in the Corsican Wetlands. Pathogens.

[55] Sgroi G., D’Alessio N., Auriemma C., Salant H., Gallo A., Riccardi M.G., Alfano F., Rea S., Scarcelli S., Ottaviano M. (2023). First Molecular Detection of *Babesia vulpes* and *Babesia capreoli* in Wild Boars from Southern Italy. Front. Vet. Sci..

[56] Ebani V.V., Bertelloni F., Cecconi G., Sgorbini M., Cerri D. (2017). Zoonotic Tick-Borne Bacteria among Wild Boars (*Sus scrofa*) in Central Italy. Asian Pac. J. Trop. Med..

[57] Díaz-Cao J.M., Adaszek Ł., Dzięgiel B., Paniagua J., Caballero-Gómez J., Winiarczyk S., Winiarczyk D., Cano-Terriza D., García-Bocanegra I. (2022). Prevalence of Selected Tick-Borne Pathogens in Wild Ungulates and Ticks in Southern Spain. Transbound. Emerg. Dis..

[58] Myczka A.W., Steiner-Bogdaszewska Ż., Filip-Hutsch K., Oloś G., Czopowicz M., Laskowski Z. (2021). Detection of *Anaplasma phagocytophilum* in Wild and Farmed Cervids in Poland. Pathogens.

[59] Polin H., Hufnagl P., Hauschmid R., Gruber F., Guther L. (2004). Molecular Evidence of *Anaplasma phagocytophilum* in *Ixodes ricinus* Ticks and Wild Animals in Austria. J. Clin. Microbiol..

